# Lipid metabolism in ferroptosis: mechanistic insights and therapeutic potential

**DOI:** 10.3389/fimmu.2025.1545339

**Published:** 2025-03-11

**Authors:** Daoyun Sun, Longfei Wang, Yufan Wu, Yi Yu, Yufeng Yao, Hongju Yang, Chunlin Hao

**Affiliations:** ^1^ Key Laboratory of Combinatorial Biosynthesis and Drug Discovery (Ministry of Education), School of Pharmaceutical Sciences, Wuhan University, Wuhan, China; ^2^ Children’s Hospital Affiliated to Zhengzhou University, Henan Children’s Hospital Zhengzhou Children’s Hospital, Henan Province Engineering Research Center of Diagnosis and Treatment of Pediatric Infection and Critical Care, Zhengzhou, Henan, China; ^3^ Key Laboratory of Molecular Biophysics of the Ministry of Education, College of Life Science and Technology and Center for Human Genome Research, Huazhong University of Science and Technology, Wuhan, China; ^4^ Division of Geriatric Gastroenterology, The First Affiliated Hospital of Kunming Medical University, Kunming, Yunnan, China; ^5^ Department of Gastroenterology, Zhongnan Hospital of Wuhan University, Wuhan, China

**Keywords:** ferroptosis, lipid peroxidation, iron metabolism, antioxidant defense, therapeutic targeting

## Abstract

Ferroptosis, an iron-dependent form of regulated cell death driven by lipid peroxidation, plays a pivotal role in various physiological and pathological processes. In this review, we summarize the core mechanisms of ferroptosis, emphasizing its intricate connections to lipid metabolism, including fatty acid synthesis, phospholipid remodeling, and oxidation dynamics. We further highlight advancements in detection technologies, such as fluorescence imaging, lipidomics, and *in vivo* PET imaging, which have deepened our understanding of ferroptotic regulation. Additionally, we discuss the role of ferroptosis in human diseases, where it acts as a double-edged sword, contributing to cancer cell death while also driving ischemia-reperfusion injury and neurodegeneration. Finally, we explore therapeutic strategies aimed at either inducing or inhibiting ferroptosis, including iron chelation, antioxidant modulation, and lipid-targeted interventions. By integrating mechanistic insights, disease relevance, and therapeutic potential, this review provides a comprehensive perspective on ferroptosis as a crucial interface between lipid metabolism and oxidative stress.

## 1 Introduction

Ferroptosis is a non-apoptotic form of regulated cell death driven by iron-dependent membrane lipid peroxidation, which compromises plasma membrane permeability and integrity, ultimately leading to membrane rupture and cell death (1–3). A defining hallmark of ferroptosis is lipid peroxidative damage, which can occur either nonenzymatically or through enzyme-catalyzed processes (4). In both cases, iron availability and the accumulation of lipid peroxides are crucial preconditions for the initiation of ferroptosis. As a self-protection mechanism, antioxidant enzymes such as glutathione peroxidase 4 (GPX4) and ferroptosis suppressor protein 1 (FSP1, also known as AIFM2) function to monitor and regulate cellular lipid peroxide levels, preventing them from reaching toxic thresholds. Inhibition of GPX4 results in a significant increase in lipid peroxidation (5–7). Conceivably, the fate of ferroptosis ultimately depends on the balance between lipid peroxidation and cellular surveillance mechanisms. Additionally, as ferroptosis is intrinsically intertwined with lipid metabolism, it is tightly regulated by cellular lipid composition, synthesis, storage, availability, and degradation. Among all lipid species, phospholipids (PLs) acylated with polyunsaturated fatty acids (PUFAs) are the most prone to lipid peroxidation, making them the primary driving force of ferroptosis (8).

Though ferroptosis is a relatively newly discovered form of regulated cell death, ferroptosis has gained considerable attention, and increasing evidence suggested that it plays a critical role in various diseases, including cancer, neurodegenerative diseases, and tissue ischemia injury (9). It has been shown that ferroptosis causes neuronal cell death and synaptic damage in Alzheimer’s disease, and its inhibition mitigates disease progression and improves cognitive function in patients (10–12). In cancer treatment, drug-resistant cancer cells can be effectively eliminated by ferroptosis-inducing compounds (13, 14). Therefore, understanding the molecular mechanism of ferroptosis and elucidating its regulatory pathways in physiological and pathological conditions hold great potentials for developing new therapeutic strategies for human diseases. In this review, we summarize the underlying mechanisms of ferroptosis, with a particular focus on its intricate connections to lipid metabolic pathways and their regulation. We highlight advancements in detection techniques and methodologies for studying ferroptosis. Furthermore, we discuss the pathogenic roles of ferroptosis in different kinds of human diseases, as well as its therapeutic potentials.

## 2 Mechanisms of ferroptosis

**Figure 1:**
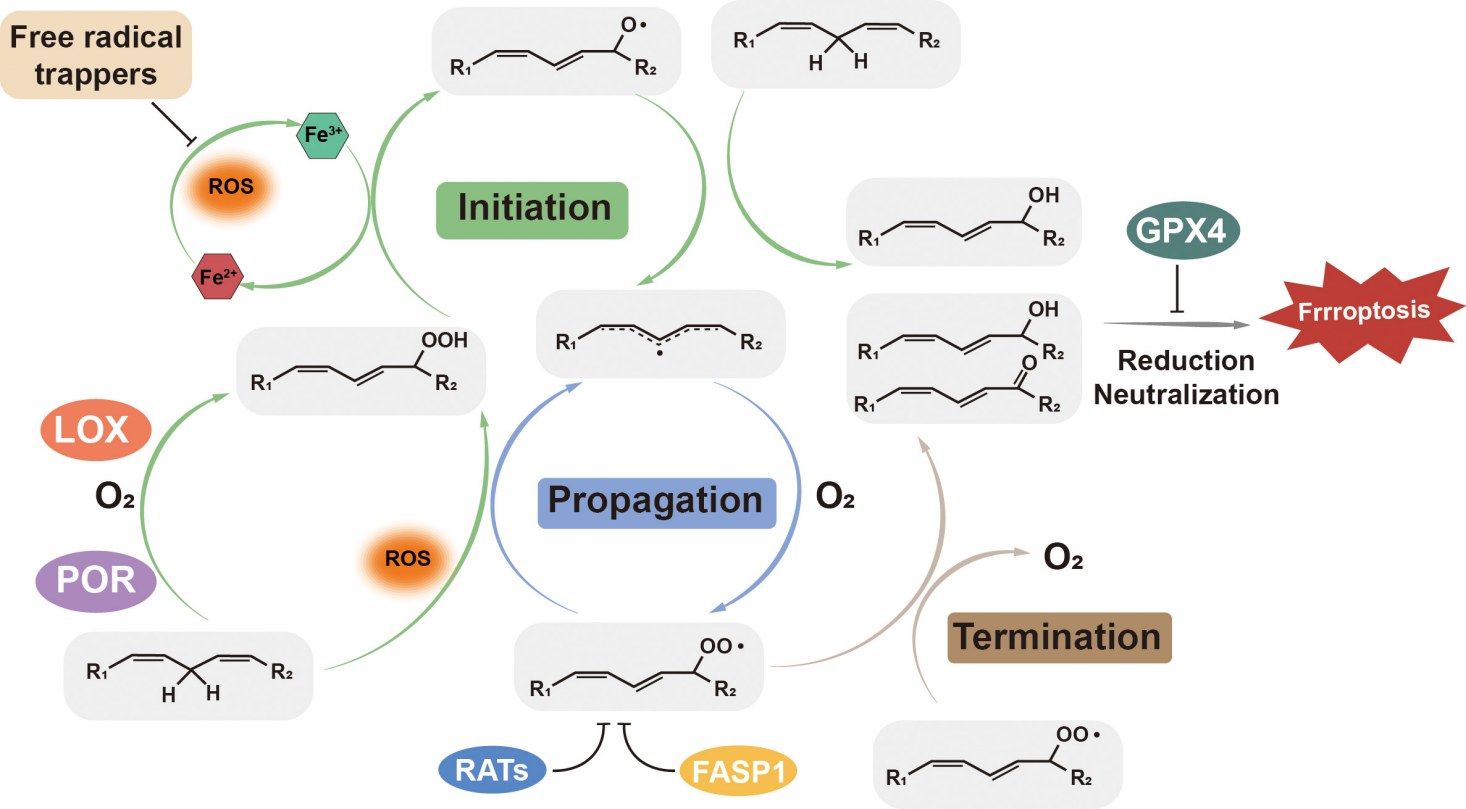
Lipid peroxidation: initiation, propagation, and termination. ROS, such as hydroxyl radicals (•OH) and peroxyl radicals (HOO•), initiate lipid peroxidation by attacking PUFA-PLs, forming lipid radicals (PLO•). These radicals propagate the reaction by reacting with oxygen to form lipid peroxyl radicals (PLOO•), which further oxidize adjacent PUFA-PLs. GPX4, Glutathione Peroxidase 4; LOX, Lipoxygenase; POR, P450 Oxidoreductase; RATs, Redox-Active Transporters; FASP1, Ferroportin 1.

Mechanistically, cellular lipid peroxidation occurs in three key steps: (1) Initiation, where reactive oxygen species (ROS) abstract a hydrogen atom from PUFAs-containing membrane phospholipids, generating lipid radicals (L^•^); (2) Propagation, where the lipid radicals react with oxygen, leading to production of lipid peroxyl radicals (LOO^•^), which further react with additional PUFA-containing phospholipids, producing lipid peroxides ((LOOH)) and propagating the lipid peroxidation chain reaction; (3) Termination, where antioxidants such as GPX4 neutralize oxidizing molecules, particularly lipid peroxyl radicals and lipid peroxides, preventing further oxidative damage (15, 16). Disruption in redox or lipid homeostasis, or deficiencies in antioxidant defense, result in an excessive accumulation of lipid peroxides, ultimately triggering ferroptotic cell death. Free intracellular iron or iron-containing enzymes serve as catalytic drivers of ferroptosis, actively participating in all three steps of lipid peroxidation (17, 18). In following subsections, we discuss iron dysregulation, lipid peroxidation, and the antioxidant defense system in detail. **Figure 1** provides an overview of the three key steps of ferroptosis, highlighting crucial enzymes and compounds involved in the process.

### 2.1 Catalytic role of iron

As indicated by its name, ferroptosis is iron-dependent, and iron plays a pivotal role in both the initiation and regulation of ferroptosis. Within cells, most iron is bound in iron-sulfur clusters or stored in ferritin, an iron storage protein, since free intracellular iron is highly reactive and can catalyze the Fenton reaction, producing highly reactive hydroxyl radicals (^•^OH). These hydroxyl radicals then react with membrane PUFAs, generating lipid radicals that initiate lipid oxidation (19, 20). Ferrous ions (Fe2+) can also interact with lipid peroxides to produce peroxyl radicals, accelerating the propagation of lipid peroxidation and promoting ferroptosis (21). Additionally, iron overload inhibits GPX4, causing the accumulation of lipid peroxides to toxic levels (22). Dysregulated iron metabolism and overload are linked to aberrant ferroptosis, contributing to the pathogenesis of various diseases (23). Q-z Tuo and colleagues have demonstrated that ischemia-reperfusion injury induces ferroptotic iron accumulation in brain by impairing the iron efflux function (24). Studies also have revealed that deletion of the gene encoding iron storage protein ferritin enhances ferroptosis in cardiovascular cells and hepatocytes (25, 26).

### 2.2 Lipid peroxidation in ferroptosis

Due to the weak double bonds present in polyunsaturated fatty acids (PUFAs), hydrogen atoms are more easily abstracted from PUFAs than from saturated or monounsaturated fatty acids. Cellular or organelle membranes are rich in phospholipids that are incorporated with PUFAs, making them highly susceptible to peroxidation. The peroxidation of PUFAs-containing phospholipids in membranes compromises membrane integrity, increases permeability, and ultimately leads to membrane rupture and cell death. Lipid peroxidation also generates toxic byproducts, including 4-hydroxynonenal (HNE) and malondialdehyde (MDA), which further damage proteins, DNA, and lipids by forming adducts with these biommolecules (16).

Lipid peroxidation occurs through either nonenzymatic or enzymatic pathways. Nonenzymatically lipid peroxidation can be initiated by hydroxyl and/or peroxyl radicals, which are products of Fenton reaction that is catalyzed by labile iron. Once lipid peroxides are formed, they propagate the peroxidation to the neighboring PUFAs-containing phospholipids with the presence of ferrous iron, and produce more lipid hydroxyl and peroxyl radicals, unless they are rapidly neutralized (**Figure 1**) (27, 28).

Alternatively, enzymatic lipid peroxidation is mediated by various iron-dependent enzymes (18). Among them, lipoxygenases (LOXs) are the most well-characterized oxygenase involved in lipid peroxidation. LOXs are a family of non-heme iron-containing dioxygenases that insert oxygen into PUFAs, producing lipid radicals. There are six different LOXs in human, each with distinct substrate selectivity and oxidation site specificity (29). For example, arachidonate 5-lipoxygenase (ALOX5) preferentially oxidizes the PUFA arachidonic acid at carbon-5, resulting in the formation of 5- hydroperoxyeicosatetraenoic acid (5-HPETE) (30). Another key enzyme family involved in lipid peroxidation is cytochrome p450 (CYPs), a group of heme-containing monooxygenases capable of directly catalyzing lipid peroxidation. CYPs accept electrons transferred by NADPH-cytochrome P450 reductase (POR) and generate ROS to initiate the peroxidation (31). However, there enzymes are not indispensable in ferroptosis, as nonenzymatic lipid peroxidation can drive the ferroptosis independently (18).

### 2.3 Antioxidant defense systems

**Figure 2:**
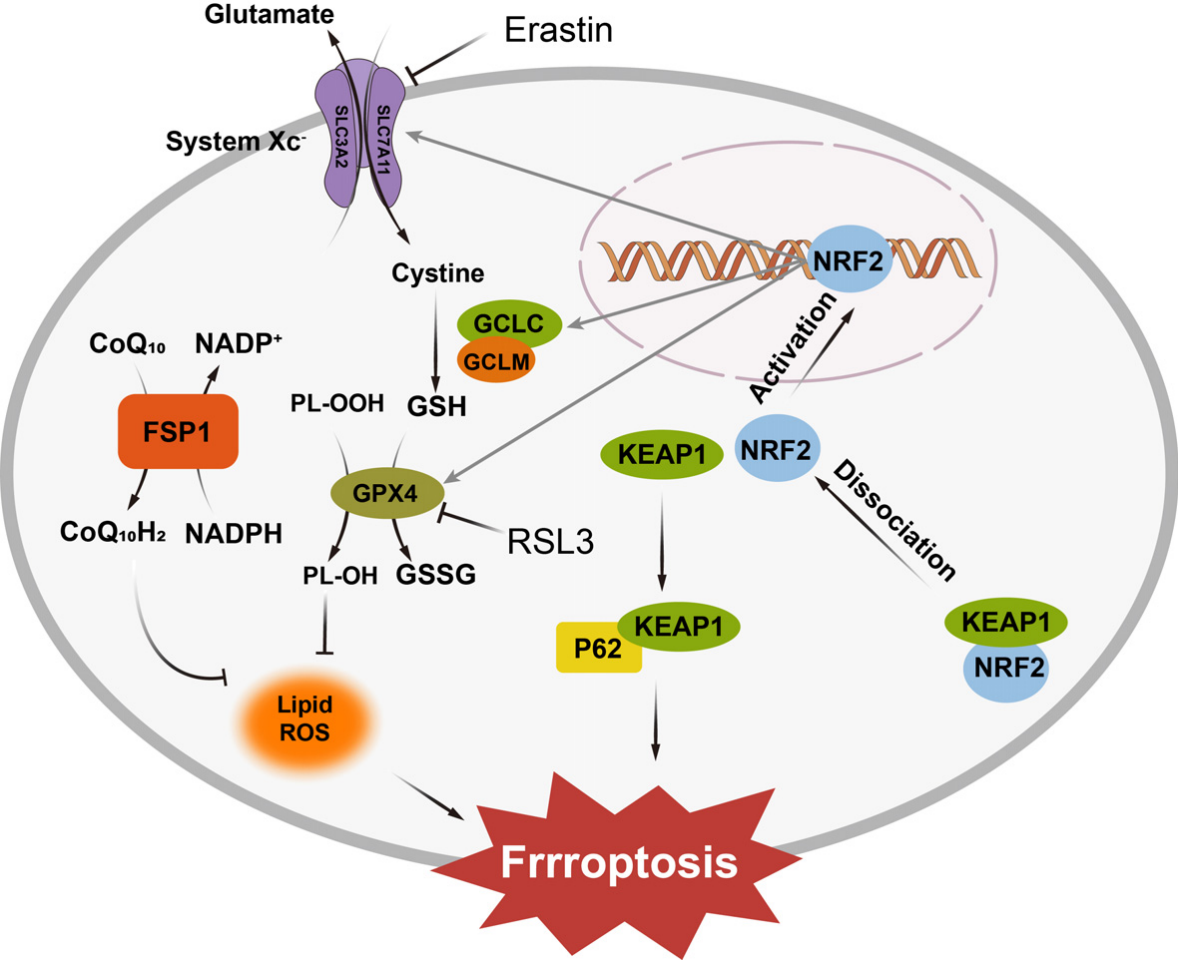
The System Xc^−^/GSH/GPX4 axis imports cystine for glutathione synthesis, with GPX4 neutralizing lipid hydroperoxides. The FSP1/CoQ10 system regenerates reduced ubiquinol to halt lipid peroxidation, while the GCH1/BH4 axis protects membranes by neutralizing lipid radicals. Under oxidative stress, Nrf2 translocates to the nucleus and activates the expression of antioxidant enzymes, including GPX4 and glutathione synthase, enhancing the cell’s ability to neutralize ROS and prevent ferroptosis. SLC3A2, Solute Carrier Family 3 Member 2; SLC7A11, Solute Carrier Family 7 Member 11; NRF2, Nuclear Factor Erythroid 2-Related Factor 2; KEAP1, Kelch-like ECH-associated Protein 1; GCLC, Glutamate-Cysteine Ligase Catalytic Subunit; GCLM, Glutamate-Cysteine Ligase Modulatory Subunit; GSH, Reduced Glutathione; GSSG, Oxidized Glutathione; GPX4, Glutathione Peroxidase 4; FSP1, Ferrostatin-1; CoQ10, Coenzyme Q10; CoQ10H2, Reduced Coenzyme Q10; NADP^+^, Nicotinamide Adenine Dinucleotide Phosphate; NADPH, Reduced Nicotinamide Adenine Dinucleotide Phosphate; RSL3, Ras-selective lethal 3; P62, Sequestosome 1.

Antioxidant defense systems play a critical role in counteracting ferroptotic stress in cell. The well-known system Xc^-^/GSH/GPX4 axis is an canonical mechanism that safeguards cells from lipid oxidation. **Figure 2** displayed the antioxidant defense systems under the ferroptotic stress. In this pawthway, cystine/glutamate antiporter (system Xc^-^) imports cystine, which is subsequently reduced to cysteine for glutathione (GSH) synthesis (29). GSH acts as a key cofactor for GPX4, the only known mammalian enzyme capable of reducing phospholipid hydroperoxides (PLOOH) into non-toxic phospholipid alcohol (PLOH), thereby preventing peroxidative damage to membranes (30–33). Disrupting this pathway—either by inhibiting system Xc^-^ with small molecule erastin or directly inactivating GPX4 with RSL3, depletes GSH, inactivates GPX4, and allows lethal LOOH accumulation (34, 35).

Beyond GPX4, the FSP1/CoQ10 system provides an independent line of defense. Ferroptosis suppressor protein 1 (FSP1) regenerates reduced ubiquinol (CoQ10H2) from oxidized ubiquinone (CoQ10) using NAD(P)H, enabling ubiquinol to act as a radical-trapping antioxidant that halts lipid peroxidation chain reactions (36). The mevalonate pathway further supports ferroptosis resistance by supplying CoQ10 precursors, linking cellular metabolism to antioxidant capacity (37).

Additionally, the GCH1/BH4 axis also contributes to lipid protection. GTP cyclohydrolase 1 (GCH1), the rate-limiting enzyme in tetrahydrobiopterin (BH4) biosynthesis, safeguards cellular membranes against ferroptosis by orchestrating antioxidant defense and lipid remodeling (38). By elevating intracellular levels of BH4 and its oxidized form BH2, GCH1 enables these metabolites to directly neutralize lipid radicals and selectively inhibit peroxidation of phospholipids. Moreover, the GCH1/BH4 axis promotes ferroptosis resistance by regenerating reduced CoQ10H2 (39, 40). The dual functionality of this pathway is evidenced by the ability of BH4/BH2 supplementation to restore cell viability under ferroptosis-inducing conditions.

Regulatory networks, such as the p62-Keap1-NRF2 pathway, integrate cellular stress signals to modulate ferroptosis sensitivity (41). Under oxidative stress, p62 sequesters Keap1, allowing nuclear factor erythroid 2-related factor 2 (NRF2) to translocate to the nucleus and activate antioxidant and iron-regulatory genes. NRF2 upregulates enzymes involved in GSH synthesis (e.g., GCLC, GCLM), GPX4, and heme oxygenase-1 (HO-1) while simultaneously enhancing iron storage (via ferritin) and export (via ferroportin) (42). This dual regulation mitigates both oxidative damage and labile iron accumulation, highlighting NRF2 as a master regulator of ferroptosis resistance.

## 3 Interconnection between ferroptosis and lipid metabolism

How cells store, remodel, and metabolize lipids can either exacerbate or mitigate lipid peroxidation, directly affecting their sensitivity to ferroptosis (16). Lipid metabolic pathways, including fatty acid synthesis, uptake, *β*-oxidation, phospholipid synthesis and remodeling, lipid storage, and release, interact with the cell’s antioxidant defenses to regulate ferroptosis sensitivity (43). These metabolic processes influence the availability of substrates for peroxidation and the capacity of cells to cope with oxidative stress. Understanding how lipid metabolism intersects with oxidative stress responses provides valuable insight into how cells balance lipid homeostasis, either promoting survival or triggering ferroptotic death in response to environmental stresses.

### 3.1 Fatty acid synthesis

The synthesis of saturated fatty acids (SFAs) and monounsaturated fatty acids (MUFAs) is primarily mediated by enzymes such as acetyl-CoA carboxylase (ACC) and fatty acid synthase (FASN). These enzymes convert acetyl-CoA into malonyl-CoA and further into palmitic acid, the SFA, which can be desaturated into MUFAs by stearoyl-CoA desaturase 1 (SCD1) (44, 45). In cancer cells, the overexpression of these enzymes supports fatty acid synthesis for energy and membrane biogenesis. However, the synthesis of SFAs and MUFAs generally confers resistance to ferroptosis, as these fatty acids are less susceptible to peroxidation than polyunsaturated fatty acids (PUFAs) (46). Conversely, the synthesis of PUFAs, which cannot be produced *de novo* in mammals, relies on dietary intake and subsequent desaturation and elongation reactions catalyzed by enzymes such as FADS1, FADS2, and ELOVL5. These PUFAs are incorporated into phospholipids, making cellular membranes more prone to ferroptosis due to their high susceptibility to lipid peroxidation (47).

### 3.2 Lipid uptake

Cells can absorb free fatty acids or lipoproteins from the extracellular environment through transporters such as CD36, fatty acid transport proteins (FATPs), and fatty acid-binding proteins (FABPs) (48, 49). CD36-mediated FA uptake can induce LPO and ferroptosis in tumor-infiltrating CD8^+^ T cells, blocking CD36 and restoring their antitumor activity (50). The cholesterol metabolite 27-hydroxycholesterol (27HC) enhances lipid uptake and metastatic capacity in aggressive breast cancer cells resistant to ferroptosis (51).

### 3.3 β-Oxidation

*β*-Oxidation is a process that breaks down fatty acids into acetyl-CoA units, primarily occurring in the mitochondria. This process is initiated by converting fatty acyl-CoA to carnitine esters by carnitine palmitoyltransferase 1 (CPT1), allowing the fatty acids to enter the mitochondria. Inside the mitochondria, fatty acyl-CoA is released from carnitine by CPT2 and undergoes a series of reactions that cleave two carbon units from the acyl chain (52, 53). The rate-limiting enzyme for *β*-oxidation of unsaturated fatty acids is 2,4-dienoyl-CoA reductase 1 (DECR1), which catalyzes the reduction of double bonds in fatty acids (54). *β*-Oxidation generally suppresses ferroptosis by reducing the availability of unesterified PUFAs, which are substrates for lipid peroxidation. In cancer cells, inhibiting *β*-oxidation can enhance ferroptosis by increasing the levels of free PUFAs and promoting lipid peroxidation (55, 56). Additionally, *β*-oxidation can influence the balance between fatty acid synthesis and degradation, affecting the overall lipid composition of cellular membranes and the susceptibility to ferroptosis (43).

### 3.4 Phospholipid synthesis and remodeling in ferroptosis

**Figure 3:**
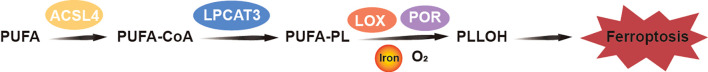
ACSL4 and LPCAT3 promote ferroptosis by facilitating the formation of polyunsaturated fatty acid phospholipids, thereby increasing susceptibility to lipid peroxidation. PUFA, Polyunsaturated Fatty Acid; ACSL4, Acyl-CoA Synthetase Long-chain Family Member 4; PUFA-CoA, Polyunsaturated Fatty Acyl-CoA; LPCAT3, Lysophosphatidylcholine Acyltransferase 3; PUFA-PL, Polyunsaturated Fatty Acid Phospholipid; LOX, Lipoxygenase; POR, P450 Oxidoreductase; PLLOH, Phospholipid Hydroperoxide.

Phospholipid synthesis and remodeling dedicates structural and oxidative properties of cellular membranes, directly influencing their ferroptosis sensitivity. Acyl-CoA synthetase long-chain family member 4 (ACSL4) and lysophosphatidylcholine acyltransferase 3 (LPCAT3) are the two main players involved in such pathways (57). ACSL4 activates long-chain PUFAs, such as arachidonic acid (AA) and adrenic acid (AdA), by conjugating them to coenzyme A (CoA) to form PUFA-CoA derivatives (58). This activation step is necessary for their subsequent incorporation into phospholipids, leading to an increase in PUFAs-containing phospholipids in membranes (50). LPCAT3 functions in parallel to ACSL4 by catalyzing the reacylation of lysophospholipids to generating PUFA-containing phospholipids, including phosphatidylcholine (PC) and phosphatidylethanolamine (PE) (59, 60). PUFA-PEs are highly reactive for LOX-driven lipid peroxidation (**Figure 3**), which triggers ferroptotic membrane destabilization greatly. Cells with a high expression profile of ACSL4 and LPCAT3 have a higher level of PUFA-containing phospholipids, predisposing them to ferroptotic damage due to the reactivity of these lipids toward oxidative stress (61). In contrast, inhibition of these enzymes diverts lipid metabolism toward monounsaturated fatty acids (MUFAs), which resist peroxidation and provide a survival advantage against ferroptotic stress (28).

### 3.5 Lipid storage

Cells store excessive fatty acids in lipid droplets in the form of triglycerides (TAGs), which can serve as a protective reservoir against lipid oxidation stress (62). Under metabolic stress, fatty acids are mobilized from lipid droplets through lipolysis, mediated by lipases, or lipophagy, a selective form of autophagy that degrades lipid droplets (63). The released fatty acids are readily available for phospholipid synthesis and remodeling, thereby alternating membrane lipid composition and ferroptosis susceptibility (64). Lipid storage in lipid droplets regulate ferroptosis in a dynamic way. In one hand, PUFAs can be stored in lipid droplets, rigorously controlling the incorporation of PUFAs to phospholipids in membranes to increase their resistance to lipid peroxidation; In the other hand, lipid droplets may facilitate the synthesis of PUFA-containing phospholipids as a PUFAs sources (37, 64).

## 4 Techniques for ferroptosis detection and analysis

Due to its complexity, a comprehensive characterization and understanding of ferroptosis require the implementation of diverse techniques across multiple scales, from subcellular structural changes to molecular alterations, lipidomic profiling, and *in vivo* validation. Microscopy and fluorescence imaging capture ferroptosis-associated morphological changes, while biochemical assays and immunodetection techniques provide insights into molecular pathways. Lipidomics and mass spectrometry-based approaches enable precise identification of oxidized phospholipids and lipid peroxidation byproducts, key markers of ferroptosis. Finally, *in vivo* models establish the physiological relevance of ferroptosis in disease contexts. **Table 1** summarizes the commonly used approaches for ferroptosis research.

**Table 1:** Tools and techniques for probing ferroptosis across subcellular, molecular, and *in vivo* levels.

Type	Approach	Target	References
Subcellular structure	Fluorescence Imaging	Membrane Integrity, Mitochondrial Viscosity	(65, 66)
	Nuclear Imaging	Nuclear Structure Integrity	(67)
	Transmission Electron Microscopy (TEM)	Mitochondrial Membrane Density, Cristae Reductions	(68)
	Immunohistochemistry (IHC)	GPX4, SLC7A11, ACSL4, KEAP1, p62	(41, 69–72)
Molecules and pathways	Endogenous Markers	ROS, Lipid Peroxidation, Iron Metabolism	(73, 74)
	Reactive Oxygen Species (ROS) Probes	Hydrogen Peroxide (H_2_O_2_), Hypochlorous Acid (HClO)	(73, 74)
	Reactivity-Based Fluorescent Probes	Labile Fe^2+^	(75)
	Lipid Peroxidation Probes	C11-BODIPY, Oxidized Phospholipids	(76, 77)
	Chemoproteomic Tools	Protein Carbonylation	(78)
	System Xc^−^/GSH/GPX4 Axis Probes	Glutathione Dynamics	(79)
	Flow Cytometry, Cell Activity Assays, Western Blotting	ROS, Lipid Metabolites (MDA), Mitochondrial Damage	(80–82)
Omics	lipidomics	Phospholipids	(83–86)
*In Vivo* Studies	Bioluminescence	Labile Fe^2+^ Levels	(87)
	Positron Emission Tomography (PET)	Ferroptosis Markers	(88)

### 4.1 Subcellular structure changes

Ferroptosis is characterized by distinct subcellular structural changes at the organelle level, particularly in mitochondria and plasma membranes, which serve as key indicators of ferroptosis. Transmission electron microscopy (TEM) has also been widely used to visualize ferroptotic morphological features, including increased mitochondrial membrane density, cristae reductions, and outer membrane shrinkage—all of which differentiate ferroptosis from other types of cell death (68). Nuclear imaging can help distinguish ferroptosis from apoptosis and necrosis, as ferroptotic cells retain nuclear integrity, whereas nuclear fragmentation or condensation is a hallmark of apoptosis and necrosis (67). Immunohistochemistry (IHC) and immunofluorescence are widely employed to detect key proteins and markers involved in ferroptosis, including GPX4, SLC7A11, ACSL4, KEAP1, and p62 (41, 70–72). These techniques provide spatial and quantitative insights into the expression and localization of ferroptosis-related proteins at both the cellular and tissue levels, offering valuable tools for studying ferroptosis’s molecular mechanisms and pathological roles.

Real-time imaging techniques further enhance ferroptosis detection by capturing membrane and organelle-specific biophysical alterations. Fluorescence imaging utilizing polarity-sensitive Mem-C1C18 and viscosity-sensitive MN-V probes allows researchers to monitor membrane integrity loss and mitochondrial viscosity shifts, providing dynamic insights into ferroptotic progression (74, 75).

### 4.2 Analyzing molecules and pathways in ferroptosis

At the molecular scale, ferroptosis is driven by reactive oxygen species (ROS), iron dysregulation, and lipid peroxidation, necessitating targeted analytical techniques for detection. Selective fluorescent probes have been developed to detect key ROS species, including hydrogen peroxide (HO) and hypochlorous acid (HClO), enabling real-time monitoring of oxidative stress within ferroptotic cells (73, 74).

Since iron catalyzes lipid peroxidation through the Fenton reaction, ferroptosis detection also involves tracking labile ferrous iron (Fe^2+^) dynamics. Reactivity-based fluorescent probes have been designed to visualize intracellular iron fluctuations, providing high-resolution insights into iron homeostasis and ferroptotic vulnerability (75).

To assess the antioxidant response and redox balance, the System Xc^−^/GSH/GPX4 axis can be monitored using fluorescence-based glutathione probes like RealThiol, which enable live-cell quantification of glutathione depletion (79).

Additionally, immunohistochemistry (IHC) and immunofluorescence are widely used to detect ferroptosis-related proteins, such as glutathione peroxidase 4 (GPX4), solute carrier family 7 member 11 (SLC7A11), acyl-CoA synthetase long-chain family member 4 (ACSL4), Kelch-like ECH-associated protein 1 (KEAP1), and sequestosome 1 (p62) (41, 69–72). These techniques provide spatial and quantitative data, offering insights into both cellular and tissue-level ferroptosis regulation.

Complementary techniques such as flow cytometry, cell viability assays, and western blotting are frequently employed to quantify ROS levels, lipid peroxidation byproducts (e.g., malondialdehyde, MDA), and mitochondrial damage, further broadening the arsenal of ferroptosis detection tools (80–82).

### 4.3 *In vivo* studies

Advanced imaging technologies, including bioluminescence and positron emission tomography (PET), have become powerful tools for detecting ferroptosis in living organisms. Bioluminescent probes like ICL-1 enable real-time, longitudinal tracking of labile ferrous iron (Fe^2+^) levels, offering dynamic insights into ferroptotic activity. Meanwhile, PET tracers such as ^18^F-TRX allow high-resolution, three-dimensional imaging of ferroptosis markers, facilitating the study of ferroptotic processes in disease models and the assessment of therapeutic interventions (87, 88).

### 4.4 lipidomics and mass-spectrometry-based approach

Lipidomics and mass spectrometry-based techniques have become essential tools for investigating ferroptosis, enabling precise characterization of lipid composition, metabolic flux, and spatial lipid remodeling. Morgan’s study demonstrated that PUFA-containing phospholipids (PUFA-PLs) determine ferroptosis susceptibility, with T cells being more vulnerable due to higher PUFA-PL levels, while myeloid cells resist ferroptosis due to lower PUFA-PL content. Wang et al. (2024) applied single-cell lipidomics to analyze lipid remodeling in foam cells, linking neutral lipid accumulation, sphingolipid depletion, and glutathione oxidation to ferroptosis progression (83).

Advanced isotope-resolved lipidomics by Reimers et al. (2023) revealed that phosphatidylethanolamines (PEs) are the primary peroxidation targets in ferroptosis (84). In an *in vivo* study, Gorman et al. (2024) employed spatial lipidomics with mass spectrometry imaging (MSI) to map iron and lipid distribution in ferroptotic ovarian tumors, showing localized accumulation of sphingolipids and triglycerides in iron-rich regions, while PUFA-containing phospholipids were enriched in peroxidized areas (85). Earlier, Doll and Kagan (2017) introduced redox phospholipidomics, identifying PEs as key substrates for lipid peroxidation upon GPX4 inhibition. Collectively, these studies highlight the power of lipidomics and mass spectrometry in deciphering ferroptotic lipid metabolism, advancing both mechanistic insights and biomarker discovery in ferroptosis research.

## 5 Ferroptosis in disease and therapeutic targeting

Ferroptosis plays a dual role in disease, acting as both a therapeutic target in cancer and a pathogenic driver in degenerative and ischemic conditions. Certain cancer cells, particularly mesenchymal-like subtypes, are highly susceptible to ferroptosis due to their elevated metabolic activity and reactive oxygen species (ROS) burden (89). Ferroptosis-inducing compounds, such as erastin, disrupt glutathione homeostasis by inhibiting system Xc^-^, leading to lethal lipid peroxidation (90). Additionally, tumor suppressor p53 sensitizes cancer cells to ferroptosis by downregulating SLC7A11, further impairing antioxidant defenses (91).

Conversely, ferroptosis contributes to tissue damage in ischemia-reperfusion (I/R) injuries, such as stroke and myocardial infarction, where excessive ROS production triggers lipid peroxidation and cell death (92). Glutamate-induced excitotoxicity inhibits system Xc^−^, driving ferroptosis in neurons during stroke, while in the heart, ferroptotic cardiomyocyte death worsens ischemic injury (93, 94). Ferroptosis is also implicated in neurodegenerative diseases, fibrosis, and autoimmune disorders, where iron dysregulation and lipid peroxidation drive cell death and disease progression (95–101).

Given its contrasting roles, therapeutic strategies targeting ferroptosis depend on disease context, aiming either to induce ferroptosis (e.g., in cancer) or inhibit it (e.g., in degenerative and ischemic diseases). Over the past decade, advances in iron metabolism regulation, antioxidant therapies, pathway modulation, and nanoparticle-based drug delivery have paved the way for clinical applications of ferroptosis-targeting strategies.

### 5.1 Regulating iron metabolism

Since iron accumulation catalyzes lipid peroxidation and ferroptosis, iron chelation therapy is widely used to prevent ferroptotic damage in conditions like thalassemia and hemochromatosis (102). Chelators such as deferoxamine (DFO), deferasirox (DFX), and deferiprone (DFP) bind excess iron, reducing oxidative stress and lipid peroxidation (103). In clinical settings, DFO has been shown to inhibit ferroptotic cardiomyocyte death, improving cardiac function in patients with iron overload, while DFX protects hepatocytes by preventing iron-driven ferroptosis (104–106).

### 5.2 Antioxidant-based ferroptosis inhibition

Since oxidative stress is central to ferroptosis, antioxidant-based therapies aim to restore redox balance. N-acetylcysteine (NAC), a glutathione (GSH) precursor, has demonstrated ferroptosis inhibition in diseases where GSH depletion exacerbates oxidative damage (107–109). Coenzyme Q10 (CoQ10), a radical-trapping antioxidant (RTA), has been studied for its protective effects in neurodegeneration, cardiovascular disease, and cancer (110). Clinical trials (NCT01964001) suggest that CoQ10 supplementation in hepatocellular carcinoma (HCC) patients improves antioxidant capacity and reduces inflammation, potentially by suppressing ferroptosis (111).

### 5.3 Targeting the SLC7A11/GPX4 axis

The SLC7A11/GPX4 pathway is a critical ferroptosis regulator, making it a key therapeutic target. GPX4 stabilizers and activators offer protection against ferroptotic damage in degenerative diseases (13, 112). Sulforaphane, a natural Nrf2 activator, enhances GPX4 expression, mitigating ferroptotic stress in various disease models (113). Clinical trials (NCT02255682) are currently evaluating CoQ10’s role in ferroptosis prevention in diabetic cardiomyopathy (114).

### 5.4 Nanoparticle-based therapies

Nanotechnology provides targeted delivery of ferroptosis modulators, enhancing therapeutic efficacy and bioavailability. Triphenylphosphine-modified quercetin nanoparticles (TQCN) selectively inhibit ferroptosis in neurons by chelating iron and activating Nrf2-mediated antioxidant responses (115). Similarly, pH/GSH-responsive polyamino acid nanogels (NG/EDA) deliver edaravone to ischemic brain tissue, preventing ferroptotic damage and improving neurological recovery (116). These nanoparticle-based strategies hold significant potential for precisely modulating ferroptosis in clinical applications.

## 6 Conclusion

Ferroptosis represents a unique intersection of lipid metabolism, oxidative stress, and iron regulation, offering fresh insights into cell death mechanisms and their implications in disease. Characterized by the iron-dependent peroxidation of polyunsaturated fatty acids (PUFAs), ferroptosis is implicated in a wide range of pathological conditions, including cancer, neurodegenerative diseases, cardiovascular disorders, and metabolic syndromes. By unraveling its molecular underpinnings—such as the roles of ACSL4, GPX4, and lipid remodeling enzymes— researchers have identified promising opportunities for therapeutic intervention.

Modulating ferroptosis presents a dual therapeutic strategy: inducing ferroptotic cell death to eliminate cancer cells or inhibiting it to protect tissues from oxidative damage in diseases driven by lipid peroxidation. Advances in dietary, pharmacological, and genetic approaches targeting lipid metabolism and iron homeostasis further underscore its therapeutic potential. However, significant challenges remain, including understanding tissue-specific ferroptotic responses and overcoming resistance mechanisms in cancer therapy (117).

Future research should aim to refine *in vivo* models, identify reliable biomarkers, and develop precise, context-dependent therapeutic strategies. Integrating lipidomics and systems biology will be critical for deciphering ferroptosis regulatory networks, paving the way for innovative treatments. Ultimately, as a regulated cell death pathway driven by iron-dependent lipid peroxidation, ferroptosis bridges lipid metabolism, redox biology, and metal homeostasis. This intersection not only redefines our understanding of cellular demise but also unveils novel therapeutic avenues for diseases ranging from cancer to neurodegeneration (35, 118).
